# Lysosomes as Dynamic Regulators of Metabolic Signaling and Organ Physiology in Aging: From Mechanism to Therapy

**DOI:** 10.14336/AD.2025.0275

**Published:** 2025-04-18

**Authors:** Yu Sun, Jin Wei, Shiyin Ma, Chang He, Liutao Sui, Xudong Pan, Xiaoyan Zhu

**Affiliations:** ^1^Department of Neurology, The Affiliated Hospital of Qingdao University, Qingdao, Shandong, China.; ^2^Department of Critical Care Medicine, The Affiliated Hospital of Qingdao University, Qingdao, Shandong, China

**Keywords:** Lysosomes, aging, senescent cells

## Abstract

Lysosomes are degradation centers and signaling hubs that in cells and play important roles in cellular homeostasis, development, and aging. Growing evidence has also implicated the role of lysosome-related mechanisms in the aging process. Meanwhile, the potential impact of lysosomal dysfunction on the production of inflammatory molecules, cellular metabolic status, and mitochondrial function is becoming increasingly significant. In this review, we provide a comprehensive overview of the physiological roles of lysosomes and their association with aging. At the cellular level, lysosomal dysfunction and cellular senescence show strong correlations. Herein, we elucidated the precise mechanisms by which lysosomal dysfunction contributes to various cellular physiological processes, as well as its potential implications in age-related hallmarks. More importantly, we discuss how lysosomal homeostasis is disrupted in several age-related diseases, including atherosclerosis, heart diseases, cancer, neurodegenerative diseases, metabolic disorders, and motor system diseases. Thus, a deeper understanding of lysosomal function may provide fundamental insights into human physiology and age-related diseases. Furthermore, these discoveries emphasize the role of the lysosome in the development of novel therapeutic strategies.

## Introduction

Aging Aging involves the decline of physiological functions, leading to diseases like cardiovascular disorders, neurodegenerative conditions, and metabolic diseases [1, 2]. A key hallmark of aging is the accumulation of senescent cells across multiple tissues. Lysosomal dysfunction, a feature commonly associated with aging and age-related diseases [3], contributes to cellular alterations such as elevated oxidative stress, the accumulation of damaged macromolecules and organelles, and enhanced lysosomal secretion [3].

Cellular senescence is traditionally characterized by the durable cessation of the cell cycle due to stress or injury, leading to numerous changes within the cell. Cellular senescence plays diverse roles in physiological and pathological processes in humans, mice, and other species [4, 5]. An increased proportion of senescent cells plays a significant role in the development of age-related diseases [2]. Senescent cells compromise cell proliferation and induce anti-apoptotic signaling pathways. The phenomenon of cellular senescence is further distinguished by alterations in chromatin architecture, adjustments in metabolism, accumulation of damaged macromolecules, and genomic instability. Senescent cells show increases in both the number and size of lysosomes. Lysosomal function further plays a pivotal role in cellular homeostasis, not only because of its recycling functions for deteriorated molecules and malfunctioning organelles, but also because of its influence on metabolic pathways and organelles such as the mitochondria [1]. SA-β-gal activity is associated with an increase in lysosomal mass [4]. Furthermore, senescent cells frequently display increased levels of senescence-associated β-galactosidase, which can be detected under acidic conditions (pH 6.0). Notably, SA-β-gal is localized in lysosomes. Moreover, it can be identified at a pH of 4 in young cells, whereas in senescent cells, it is observed at a pH level of 6 [6]. Limited attention towards the enzyme's activity at elevated pH in senescent cells has only recently been sparked following the identification of lysosomal impairment and changes in acidification within senescent cells [7, 8]. Senescence often coincides with additional traits that may be associated with lysosomal function, including mitochondrial dysfunction, impairment of protein homeostasis, abnormal regulation of nutrient sensing, and altered intercellular communication [1].

Reduced lysosomal activity serves as a characteristic feature of several physiological states, such as aging and neurodegeneration, while its overactivation is a defining feature in specific types of cancers. These results indicate that dysfunctional lysosomes contribute to the development of several diseases. As such, significant effort has been devoted to identifying innovative strategies for manipulating lysosomal function as potential therapeutic interventions for these pathological states [9]. Lysosomes have further attracted considerable interest in diverse disciplines owing to their ability to regulate energy metabolism, nutritional condition, and cellular quality control mechanisms.

Currently, the exploration of lysosomal dysfunction in the context of aging and aging-related diseases remains incomplete. Moreover, there are ongoing debates and a lack of thorough, detailed summaries regarding the targeting of lysosomes as a therapeutic strategy for aging-related conditions. The purpose of this review is to highlight comprehensive and novel insights into the function and regulation of lysosomes and their pathophysiological mechanisms in aging and aging-related diseases, thereby providing a solid theoretical basis for targeting lysosomes for the treatment of aging-related diseases. Furthermore, we discuss the controversies and challenges of lysosomal targeting therapy. We believe that it would be justifiable to conduct extensive investigations aimed at targeting lysosomes for therapeutic purposes.

### The Composition and Functions of lysosomes

Lysosomes are versatile, membrane-bound organelles that display diversity in their location, structure, dimensions, enzymatic content, and target specificity [10]. Lysosomes are enclosed by a lipid bilayer and house an acidic interior that facilitates breakdown processes. Within this lumen, around 60 hydrolases are present, which function optimally under acidic conditions. These enzymes play a crucial role in facilitating step-by-step catabolic processes that lead to the breakdown of organelles and macromolecules into smaller units [11]. Moreover, multiple ion channels regulate bidirectional ion flux, and lysosome-associated membrane proteins (e.g., LAMP1-5) play critical roles in diverse cellular functions such as macroautophagy, phagocytosis, lipid trafficking, chaperone-mediated autophagy (CMA), and aging-related mechanisms [12-14].

Lysosomes play a crucial role in the breakdown of proteins by housing enzymes like cathepsins. Additionally, they are actively involved in breaking down other large molecules such as lipids through the action of lysosomal acid lipase LIPA [15]. The membrane proteins play crucial roles in diverse lysosomal processes, encompassing lysosomal reformation, fusion, fission, import of sorting machinery, the formation and maintenance of lysosomal lumen PH, and the recycling of lysosomal degradation products [16]. Lysosomes play essential functions in various cellular processes beyond their primary function as recycling containers, acting as crucial signaling centres for energy and amino acid detection, signal transmission, and autophagy [17]. Beyond their degradative role, lysosomes also participate in bidirectional homeostatic crosstalk with other organelles, including mitochondria and the endoplasmic reticulum (ER) [18, 19]. Lysosomes further play a role in plasma membrane excretion and restoration [20], and contribute significantly to the development of tumors, immune reactions, and various other physiological and pathological processes [21]. As such, lysosomal dysfunction is a common cause of many human diseases.

### The Regulation of lysosomes

**Figure 1.**
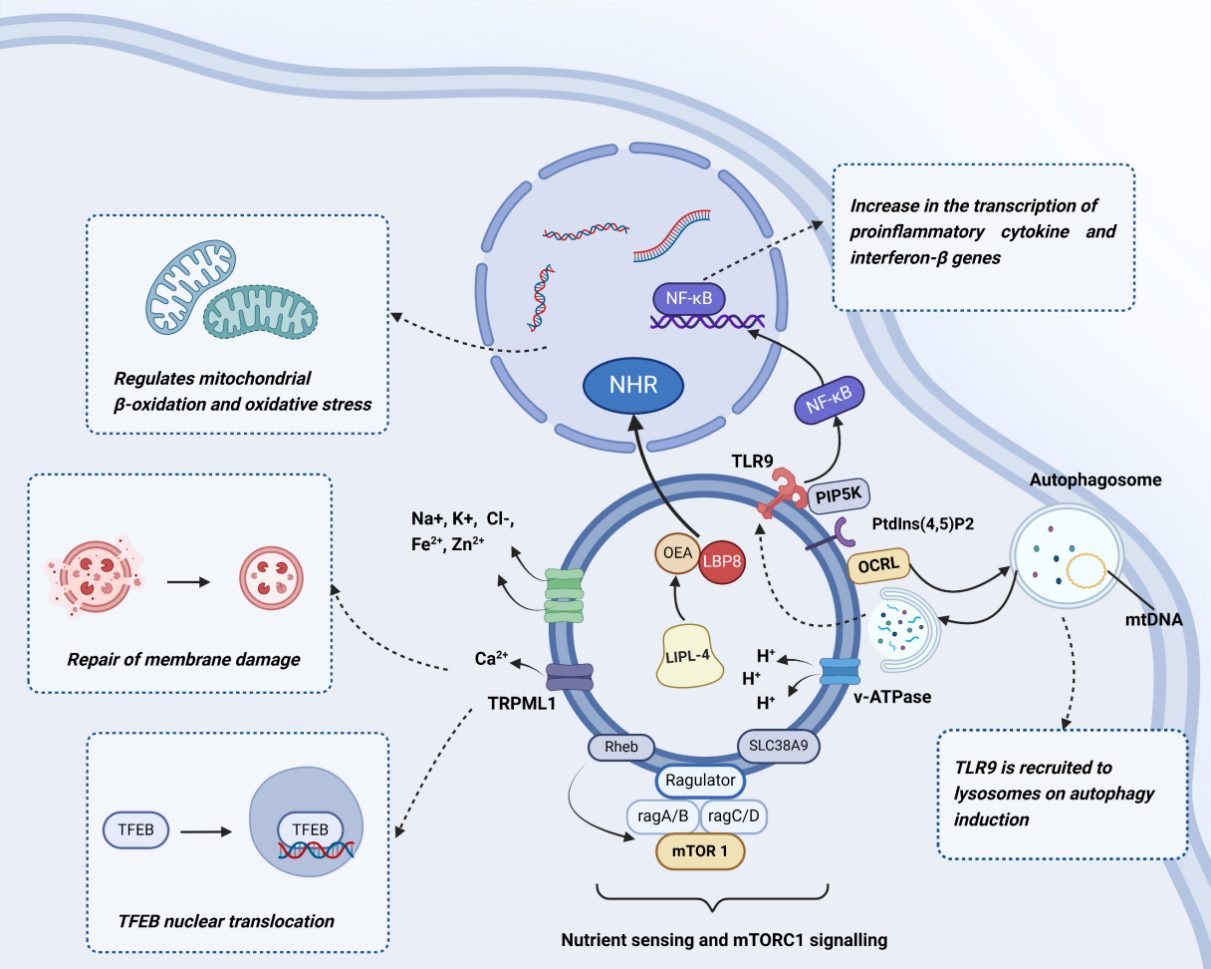
**Regulation of lysosomes**. We show some specific types of lysosomal signaling pathways. The presence of mTOR on the surface of lysosomes indicates that lysosomes play a crucial role as signalling centres within cells; The TFEB/TFE3 pathway mediates the synthesis of new lysosomes through the transcriptional upregulation of lysosomal genes; Lysosomes are crucial for mediating lipid-dependent signalling pathways; The interior of the lysosome contains a variety of ions. Created with BioRender.com.

Lysosomes play a pivotal role in degradation, innate and adaptive immunity, and nutrient sensing. Intriguing research has shed light on numerous signaling pathways that interact with lysosomal activities, thereby highlighting the central significance of lysosomes in various physiological processes [17, 22]. In this context, we will concentrate on particular lysosomal signaling pathways that react to various environmental cues and play crucial roles in regulating multiple functions, ultimately ensuring cellular homeostasis. Some specific types of lysosomal signaling pathways are shown in Figure 1.

### Lysosomal nutrient sensing and mTORC1 signaling

A major scientific advancement was the discovery that the lysosomal membrane serves as a platform for mTORC1 activation, which is triggered by nutrient sensing and growth factor signaling [23]. The primary role of mTORC1 is to facilitate cellular anabolic processes and promote growth when nutrients and growth factors are available. At the same time, it inhibits catabolic processes like autophagy by phosphorylating ULK1. Notably, mTORC1 additionally controls lysosomal reconstitution during the process of autophagy, a vital mechanism that facilitates the restoration of fully operational lysosomes when subjected to extended periods of nutrient deprivation [24]. The translocation of mTORC1 to lysosomal membranes represents a critical step in its activation mechanism, mediated by amino acid-induced Ragulator-RAG GTPase interactions [25-31]. The attraction of mTORC1 to the lysosomal membrane, in a manner dependent on RAG, can be triggered by cholesterol owing to its interaction with the NPC1, facilitating cholesterol binding.

The presence of mTOR on the surface of lysosomes indicates that lysosomes play a crucial role as signaling centres within cells [26, 32]. Recruitment of the mTORC1 complex to lysosomes occurs through a heterodimeric complex consisting of GTP-bound RAGA/B and GDP-bound RAGC/D, leading to its activation by the Rheb GTPase [27, 33, 34]. The recruitment of mTORC1 to the lysosome and its subsequent activation are facilitated by additional nutrients, including cholesterol. The regulation of mTORC1 by cholesterol involves SLC38A9 in a way that is separate from its functions in arginine sensing and amino acid transportation [35, 36]. Involvement of the lysosomal membrane in glucose and energy sensing has further been suggested. For example, when faced with a lack of glucose, the recruitment of AMPK, which serves as the primary energy sensor, to the lysosomal membrane for activation is associated with the V-ATPase and Ragulator complexes [37, 38]. AMPK is essential to control cellular metabolism. When glucose is unavailable, axin, a scaffolding protein that can inhibit Ragulator activity, initiates the separation and deactivation of mTORC1 on the lysosomal membrane [39]. Furthermore, axin facilitates the recruitment of AMPK through its interaction with liver kinase B1 (LKB1), leading to the activation of AMPK via the formation of complexes involving V-ATPase and Ragulator [40]. Stimulation of AMPK signaling further improves cellular energy storage mechanisms by augmenting glucose absorption, encouraging autophagy, and impeding catabolism by interfering with mTORC1 activation.

The TFEB/TFE3 pathway regulates lysosomal biogenesis by transcriptionally upregulating lysosomal genes [41]. TFEB/TFE3 activation involves de-phosphorylation and subsequent nuclear translocation, enhancing the transcriptional control of macroautophagy and lysosomal formation. One crucial mechanism regulating the subcellular localization of TFEB/TFE3 involves mTORC1-mediated phosphorylation, which sequesters these factors in the cytosol [41] [42]. Even when canonical mTORC1 signaling is hyperactivated as is observed in senescent cells [43, 44], TFEB can still be highly activated to induce profound lysosomal expansion [45, 46]. Thus, modification of the mTORC1 signaling pathway as a reaction to many cellular stresses results in the upregulation of macroautophagy, lysosomal biogenesis, and V-ATPase-mediated lysosomal acidification. Dysregulated mTORC1 signaling has profound implications for tissue and organ growth as well as homeostasis and contributes to the development of many age-related disorders [23, 47]. Table 1 shows the functions of mTOR in several diseases.

**Table 1:** Functions of mTOR in several diseases.

Diseases	Mechanism	Function	References
**LSDs**	mTOR activation	Suppresses autophagy Suppresses TFEB	[48, 49]
**NSCLC**	Activation of AKT/mTOR signaling	Cell proliferation, and invasion	[50]
**Lung carcinoma**	Mutations of mTOR complexes	Bad prognosis and short survival	[51]
**Endometrial carcinoma**	Activation of PI3K/AKT/mTOR axis	Enhanced endometrial carcinogenesis	[52]
**TSC**	Hyperactivation of mTORC1	Epileptic seizures	[53]
**AD**	mTOR activation	Increased autophagy	[54]
**PD**	mTOR activation	Increased autophagy	[55]
**Type 2 diabetes**	mTOR activation	Activation of adipogenic/lipogenic factors	[56]
		Promotes insulin resistance	
**Dilated cardiomyopathy**	Deletion of mTORC1 components	Suppresses protein synthesis Suppresses cellular growth	[57, 58]
**Heart failure**	mTOR activation	Strong suppression of protein translation	[57]
**NAFLD**	Elevated hepatic mTORC1	Lipogenesis active	[59]

Abbreviations: NSCLC: non-small cell lung carcinoma TSC: tuberous sclerosis complex NAFLD: Non-alcoholic fatty liver disease

### Lysosomal fat-to-neuron lipid signaling pathway

Lysosomes play a crucial role as cellular organelles in the metabolism of both extracellular and intracellular substrates. The dysregulation of lysosomal metabolism has been implicated in age-related metabolic disorders and neurodegenerative diseases. Lysosomes are crucial for maintaining lipid homeostasis and mediating lipid-dependent signaling pathways [15]. Phosphoinositides, an essential lipid family integrated into lysosomal biology, play an important role in regulating diverse elements of the dynamics and functionality of lysosomes. These tasks encompass the regulation of its placement, facilitation of cellular self-generation, encouragement of merging with autophagosomes, and support of lipid exchange at contact points between the membranes [60]. A previous study elucidated the lipid signaling pathway triggered by lysosomal metabolism, as well as its role in enhancing longevity in *Caenorhabditis elegans*. The promotion of longevity is facilitated by the activation of lysosomal lipolysis in peripheral adipose tissue, leading to an enhancement of the neuropeptide signaling pathway within the nervous system [61]. Lysosomes further serve as a central signaling hub for coordinating metabolism and aging, facilitating inter-tissue communication through lysosomal signaling to promote longevity [61].

Lysosomes play a crucial role in lipid metabolism, while lysosomal acid lipases facilitate the hydrolysis of triacylglycerols (TAGs) and cholesteryl esters (CEs) to release free fatty acids (FFA). In addition, lysosomes function as central signaling hubs within the cell. In *Caenorhabditis elegans*, LIPL-4 is specifically expressed in the intestinal tissue, serving as a peripheral fat storage site. Its expression is upregulated during fasting and in long-lived mutants that exhibit reduced insulin/IGF-1 signaling or lack of a germline [62-64]. One study conducted by Savini et al. demonstrated that lysosomal lipolysis in peripheral adipose depots generates PUFAs. PUFAs, along with the lipid chaperone LBP-3, initiate a nuclear hormone receptor- and neuropeptide-mediated cascade in neurones, thereby promoting lifespan extension [61]. Recent investigations conducted on *Caenorhabditis elegans* have further demonstrated the involvement of lysosomes in signalling pathways associated with an extended lifespan, facilitated by the lipid OEA and the protein LBP8, which binds to lipids [65].

### Lysosomal ion balance regulation

The lysosomal interior contains a variety of ions, including calcium (Ca²^+^), hydrogen (H^+^), chloride (Cl^-^), sodium (Na^+^), iron (Fe²^+^), potassium (K^+^), and zinc (Zn²^+^). Lysosomal membrane channels and transporters work in concert to maintain the osmolarity within the lysosome and regulate the tension of its membrane by coordinating the movement of organic molecules, ions, and water [66, 67]. The lumen of lysosomes serves as a repository for various essential minerals, including Zn^2+^, Ca^2+^, Na^+^, and Fe^2+^ [68]. Calcium ions within lysosomes (Ca²^+^) are crucial regulators of diverse lysosomal functions. Specifically, Ca²^+^ efflux mediates the fusion between lysosomes and other cellular structures, including endosomes, autophagosomes, and the plasma membrane. This process effectively regulates the trafficking of endocytic membranes and autophagy, and repairs damage to the cell membrane [69, 70].

Over the past ten years, researchers have successfully utilized the patch-clamp technique to study lysosomes in isolation, leading to the discovery of lysosome-specific ion channels. These advancements have significantly improved our understanding of how ion channel activity is related to lysosomal dysfunction and subsequent diseases. Lysosomal Ca^2+^ channels are responsive to various stimuli, indicating that their activities can be modulated differently depending on cellular conditions, enabling more targeted Ca^2+^ signaling responses tailored specifically to distinct cellular needs [25]. Previous studies have shown that the relationship involving mitochondria and lysosomes plays a crucial role in the control of intracellular calcium levels. The release of calcium from lysosomes via the TRPML1 channels leads to an influx of calcium into the mitochondria, particularly at sites where the mitochondria and lysosomes come into contact. Furthermore, this increase in mitochondrial calcium is influenced by VDAC1 on the outer mitochondrial membrane and MCU on the inner mitochondrial membrane [71].

Maintaining ionic equilibrium across the lysosomal membrane requires compensatory potassium ion (K^+^) influx to offset the efflux of sodium (Na^+^) and calcium (Ca²^+^) ions. This crucial ion movement plays a vital role in preserving the lysosomal membrane potential, underscoring the significance of potassium channels in sustaining lysosomal function. Until now, two potassium channels have been identified within lysosomes: TMEM175 and BK. While BK functions similarly to plasma membrane-bound BK channels, TMEM175 represents a distinct type of non-canonical K^+^ channel exclusive to lysosomes [72]. In contrast to conventional potassium channels, TMEM175 exhibits distinct pharmacological properties—remaining insensitive to tetraethylammonium and quinine blockade while demonstrating zinc-dependent inhibition. TMEM175 deficiency leads to an increased rate of fusion between autophagosomes and lysosomes in RAW246.7 macrophage cells [73]. Impaired degradation of lysosomes, compromised clearance of autophagosomes through lysosomes, and decreased mitochondrial respiratory capacity have all been observed in a neuroblastoma cell model with TMEM175 knockout, which further demonstrated an impaired ability to effectively clear phosphorylated α-synuclein fibrils, which are commonly observed in individuals with Parkinson's [74].

Each individual ion channel plays a role in the maintenance of lysosomal equilibrium, although its impact on lysosomal function may vary. The CLC7Hþ/Cl exchanger is responsible for regulating chloride ions (Cl^-^), which have been found to play a part in the acidification process within lysosomes [75]; however, the results of another study indicated that lysosomes derived from CLC-7 knockout mice exhibit normal pH levels [76]. Demonstrating that CLC-7-mediated chloride transport is necessary for lysosomal acidification is crucial to validate this channel's role in lysosomal impairment. Additionally, lysosomes function as reservoirs for redox-active metals like copper and iron, maintaining their luminal concentrations at micromolar levels. Carrier proteins such as transferrin and ferritin facilitate the transportation of iron in its ferric form (Fe3^+^) to the lysosome through processes such as endocytosis and autophagy-mediated capture [77, 78]. Copper further acts as a necessary component for various kinases and respiratory enzymes. When cytoplasmic copper levels become elevated, the copper-transporting ATPase ATP7B translocates from the trans-Golgi network to lysosomes, enabling temporary sequestration of excess copper in these organelles [79].

### Lysosomal cGAS/STING pathway

The enzyme cyclic GMP-AMP synthase (cGAS) detects cytosolic double-stranded DNA (dsDNA) following microbial infection or cellular injury, initiating cGAS-STING-mediated signaling cascades. This pathway orchestrates critical biological functions such as IFN/cytokine secretion, autophagic regulation, metabolic control, and cell fate decisions (including senescence and death). As a key modulator of immune defense and tissue integrity, cGAS-STING activation must be precisely regulated—its dysregulation is implicated in diverse pathologies ranging from infectious and autoimmune diseases to chronic inflammation, neurodegeneration, and malignant transformation [80]. Lysosomal membrane permeabilisation (LMP), which can be triggered by various factors such as cathepsins, results in the release of lysosomal contents into the cytoplasm, causing cellular demise via the lysosomal cell death (LCD) pathway [81-84]. Lysosomes possess the ability to recruit endosomal sorting complexes essential for transport complex, which aids in the mending of slight impairments to the lysosomal membrane. Alternatively, if this repair mechanism fails, it can lead to the initiation of various forms of cellular demise known as lysophagy [85]. STING triggers lytic cell death (LCD) in human myeloid cells through an AIM2-independent pathway, mediated instead by NLRP3 inflammasome activation following potassium efflux [86]. STING-induced LCD appeared to play a crucial role in determining the fate of blood cells.

The cGAS/STING pathway, a key innate immune signaling cascade, is activated by severe cellular stressors like genomic instability and significantly upregulates autophagic activity [87-92]. In the event of mitotic catastrophe, dysfunctional telomeres can produce cytosolic chromatin fragments that activate cGAS-STING signaling, leading to autophagic cell death in human lung fibroblasts and epithelial cells [93]. *Burkholderia pseudomallei* can trigger cell fusion via its type VI secretion system 5 (T6SS5), generating micronuclei that activates the cGAS-STING pathway in RAW264.7 macrophages. Notably, infected cells experience autophagic cell death independent of interferon production [94]. The cGAS-STING signaling axis can initiate autophagic cell death, functioning as a host defense mechanism to limit the spread of compromised cells.

**Figure 2.**
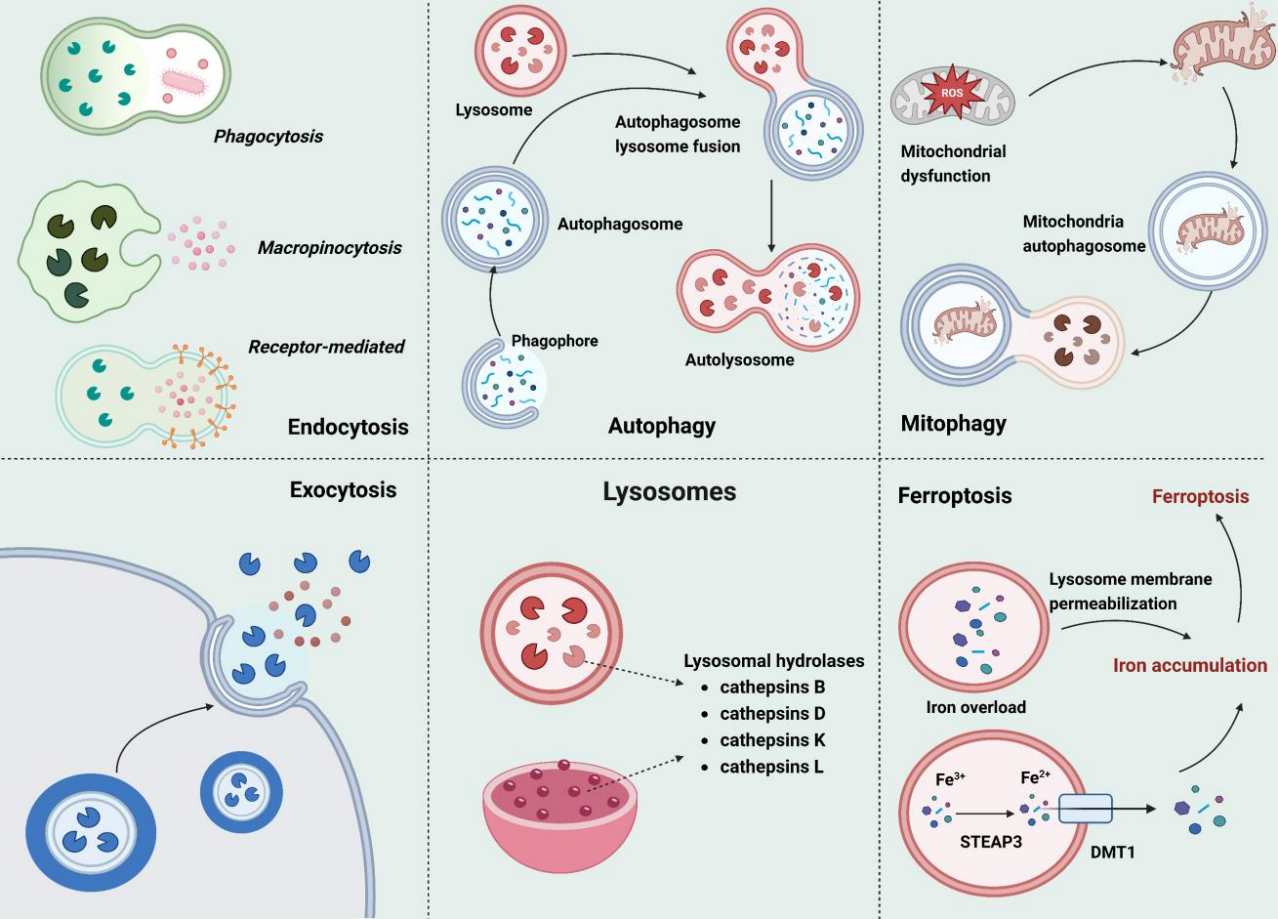
**Processes in which the Lysosome Participates**. (1) Endocytosis; (2) Autophagy; (3) Mitophagy; (4) Exocytosis; (5) Ferroptosis; Created with BioRender.com.

In addition to inducing autophagy, the cGAS/STING pathway plays an essential role in promoting the SASP, a significant characteristic of cell senescence. This process is mediated through the upregulation of interferons and pro-inflammatory cytokines via IRF3 and NF-κB signaling [93, 95-97]. Recently, the role of cGAS in detecting cytosolic DNA of the derepressed endogenous retrovirus HERVK in senescent human cells, a phenomenon not observed in early passage non-senescent cells [98], was discovered. Following cGAMP binding, STING relocates from the endoplasmic reticulum (ER) to Golgi-derived perinuclear vesicles. This movement initiates TBK1/IRF3- and NF-κB-dependent signaling cascades, culminating in type I interferon and pro-inflammatory cytokine production [93, 95-97, 99]

## Processes in which the Lysosome Participates

### Endocytosis

Lysosomes are essential in multiple processes. In Figure 2, we have illustrated several physiological processes involving lysosomes. Multiple concurrent pathways of endocytosis exist, but can all be broadly classified as clathrin-dependent or clathrin-independent. This classification is based on the presence of an essential clathrin coat protein in the endocytic machinery. Among these clathrin-mediated endocytosis (CME) has been extensively studied, and is well-understood [100]. The recruitment of clathrin to endocytic sites is facilitated by adaptor protein (AP) complexes, which aid in the inclusion of transmembrane proteins (cargo) with sorting motifs in their cytoplasmic tails into vesicles [101].

Endocytosis involves the engulfment of the cellular membrane, enabling the internalisation of extracellular materials. Studies indicate that endocytic activity progressively diminishes during aging and cellular senescence, accompanied by the downregulation of key regulators such as βPIX and GIT. βPIX, a p21-activated kinase, plays a critical role in membrane ruffle formation. Its depletion in human dermal fibroblasts triggers senescence, a phenotype characterized by impaired clathrin-mediated endocytosis. This defect correlates with amphiphysin 1 fragmentation—an essential adaptor protein that facilitates actin polymerization during endocytosis. This impaired endocytosis appears to be associated with the continuous activation of integrin signaling, which is essential for the formation of a senescent phenotype, as evidenced by its mitigation through the inhibition of integrin signaling [102].

While total endocytic rates decline during aging, caveolin-mediated endocytosis is upregulated in senescent cells. This upregulation is particularly evident in neurons, where it facilitates the prion-like propagation of pathological α-synuclein oligomers [103], a critical mechanism in the progression of synucleinopathies such as Parkinson's disease [104]. The LC3-associated phagocytosis (LAP) and endocytosis (LANDO) pathways, which play crucial roles in transporting lysosomal substrates, are also increasingly recognized as being affected by aging and age-related conditions [105-107]. The activities of most other lysosomal delivery pathways also decline with age.

### Autophagy and Mitophagy

Lysosomal degradation can be categorized into endocytosis and autophagy, which include macro-autophagy, microautophagy, and CMA. The recycling of cytoplasmic materials such as mitochondria occurs through macroautophagy initiated by the ULK and PI3K complexes. This procedure entails the creation of double-membrane phagophores, which subsequently mature into autophagosomes [108]. Autophagy is a well-preserved process that is essential for maintaining cellular homeostasis, facilitating stress relief, and preventing the onset of various diseases [109]. The targeted disruption of Atg-related genes results in embryonic death during the 4-8 cell stage, resulting the degeneration of nerve cells, deterioration of axons, enlargement and dysfunction of the liver, abnormal growth and impaired function of the heart, disrupted structure of mitochondria, shrinkage of fast muscle fibres resulting in muscle wasting, reduction in white adipose tissue, compromised mass and functionality of β-cells in the pancreas, decreased levels of T and B cells leading to lymphopenia, severe blood deficiency causing anemia, development of glomerulosclerosis at a later stage in life, and diminished amino acid concentrations; all of which are characteristics commonly observed throughout the aging process [110]. Autophagy is indispensable for preventing aging, and maintaining or restoring youthful levels of autophagy-related genes can counteract age-related damage and decline. Aging cells undergo multiple alterations, including the buildup of dysfunctional organelles and misfolded proteins, telomere attrition, genomic instability, and oxidative damage. These interrelated changes form a complex network with autophagy, collectively driving organismal aging, chronic inflammation, and senescence [111].

mTORC1, a key regulator of macroautophagy, directly phosphorylates and inhibits autophagy-initiating proteins [112, 113]. The inhibition of mTORC1-dependent nutrient signaling represents a crucial mechanism for macroautophagy upregulation, as extensively reviewed in recent literature [114, 115]. Conversely, the activation of mTORC1 is widely thought to be a significant contributing factor to the age-related decline in macroautophagic flux [115]. Additionally, a decrease in the transcriptional expression of diverse macroautophagy components is also evident during the aging process [116], and likely stems from the age-related decline in autophagy-inducing transcription factors concurrent with the epigenetic alterations affecting macroautophagy genes [117, 118]. The expression of LAMP2A, which plays a crucial role in facilitating substrate translocation into lysosomes during chaperone-mediated autophagy, also declines with age [119]. The reversal of autophagy loss in aging effectively mitigates age-related disorders, reduces endoplasmic reticulum (ER) stress, and enhances glucose tolerance [120]. The normalization of LAMP2A levels during aging also results in a decrease in the buildup of protein aggregates, marked by polyubiquitination, oxidized proteins, and programmed cell death [121]. Tissue-specific overexpression of Atg7 in liver tissue is sufficient to ameliorate autophagic impairments resulting from suppressed Atg7 expression, such as endoplasmic reticulum stress and insulin resistance. However, these advantageous outcomes are nullified when the downstream mediators of Atg7 and Atg5 are obstructed [120]. Research conducted on yeasts, worms, flies, and mice has consistently shown that the upregulation of autophagy-related genes effectively mitigates the aging process by significantly prolonging lifespan [122].

Aging is marked by the build-up of damaged mitochondria, which exhibits decreased effectiveness in generating ATP and heightened reactive oxygen species (ROS) generation [123]. Increased ROS levels further facilitate the oxidation of cellular constituents such as RNA, proteins, DNA, and lipids, leading to the initiation of oxidative stress [124]. Autophagy regulates mitochondrial health by facilitating damaged mitochondria through mitophagy [111]. The ability of lysosomes to regulate bioenergetic and biosynthetic capabilities is strongly linked to their capacity to sort mitochondria and eliminate damaged organelles via mitophagy. By working together with autophagosomes, which enclose dysfunctional mitochondria, lysosomes facilitate the degradation of these impaired organelles within their lumen [125, 126]. This procedure hinders the build-up of malfunctioning mitochondria and enhances their well-being [127].

### Ferroptosis

Ferroptosis, a newly characterized form of regulated cell demise (RCD), exhibits distinct morphological, genetic, and biochemical characteristics compared to other forms of cellular death [128]. Lysosomes contain hydrolases, such as members of the cathepsin family, which aid in the breakdown and reutilization of vital nutrients to maintain homeostasis via diverse mechanisms such as ferroptosis [129]. Mounting evidence has further substantiated the involvement of lysosomal activity in the initiation of ferroptosis [130-133]. Most cells acquire iron via endocytosis, wherein transferrin (TF), loaded with ferric iron, acts as the principal protein for transporting iron [134]. This process encompasses the interplay between TF and the transferrin receptor (TFRC, alternatively known as TFR1) on the cellular surface, which is followed by the creation of endocytic vesicles and their subsequent amalgamation with lysosomes. In the acidic environment found in lysosomes, metalloreductases, such as STEAP3, facilitate the release and reduction of ferric iron to ferrous iron. Subsequently, ferrous iron is transported to the cytosol via DMT1 or MCOLN1 [135, 136]. Ferroptosis is defined as a controlled form of cellular death triggered by the presence of intracellular iron, which stimulates ROS generation. Lysosomes play a significant role in ferroptosis, as they serve as primary storage sites for iron. The disruption of lysosomal membranes appears to facilitate the initiation of this particular pathway, leading to cell death [130]. Another pathway that plays a role in ferroptosis is observed in the lysosomes. CMA, a cellular mechanism involved in lysosome-mediated degradation, also contributes to ferroptosis [128, 133].

Dysregulation of iron homeostasis has been documented in numerous studies, either at the systemic level, or within specific organ systems influenced by age-related conditions [137-139]. There have been several separate reports on the occurrence of age-related iron buildup in different tissues, which is distinct from the enrichment observed in senescent cells [140-144]. Impaired ferritinophagy, which is responsible for ferritin degradation and ferroptosis, leads to iron accumulation in senescent cells. Lysosomal malfunction in aged cells has further been verified by various indicators such as the buildup of the microtubule-associated protein LC3-II within autophagosomes. The accumulation of iron in senescent cells can further be attributed to the impaired breakdown of ferritin, resulting in the entrapment of iron within ferritin and giving rise to a perceived cellular insufficiency. Consequently, senescent cells exhibit a high resistance to ferroptosis. Our prior research demonstrated that rapamycin-mediated autophagy activation efficiently attenuated iron accumulation in senescent cells, concurrently suppressing TfR1 upregulation, ferritin elevation, and intracellular iron overload. However, these interventions failed to reestablish ferroptotic sensitivity in these cells [145].

### Exocytosis

Lysosomes participate in secretory vesicle regulation via lysosomal exocytosis, a two-stage mechanism comprising: (1) peripheral translocation and subsequent plasma membrane fusion, followed by (2) extracellular content discharge. This process is vital for multiple physiological functions, such as membrane repair, immune activation, bone resorption, and intercellular communication [146]. Plasma membrane damage disrupts cellular compartmentalization, permitting uncontrolled bidirectional flux of molecules that induces apoptotic pathways. Rapid membrane resealing is therefore critical for maintaining cellular viability. During lysosomal exocytosis, free lysosomes are depleted through fusion with the plasma membrane and subsequent extracellular release of their cargo. This fundamental mechanism supports diverse physiological and pathological functions including wound repair, immunoregulation, and metastatic progression [147-149].

Lysosomes are mainly located in the perinuclear region. When the plasma membrane is damaged, lysosomes move to the cell edge to fuse with the plasma membrane and help repair it. This process is regulated by calcium release from lysosomes via TRPML1. Lysosomal exocytosis is often studied by detecting lysosomal proteins like LAMP1 and TRPML1 on the cell membrane [150, 151]. The ability of cells to sense increases in intracellular Ca^2+^ levels and initiate lysosomal exocytosis is one of the many mechanisms through which they can adapt to different effector stimuli [152]. The lysosomal membrane detects Ca^2+^ influx following damage to the plasma membrane through synaptotagmin-VII (Syt-VII) [152]. Following this, Syt-VII engages with the trans-SNARE complex situated on the plasma membrane. This complex is created through the interaction of VAMP7 localised within lysosomes with syntaxin-4 and SNAP23. To promote fusion, the trans-SNARE complex enables the close proximity between lysosomes and the PM. The application of ionomycin significantly suppresses lysosomal exocytosis in embryonic fibroblasts obtained from Syt-VII knockout mice expressing a dominant-negative VAMP7 variant [153]. This finding underscores the essential contributions of the trans-SNARE complex and Syt-VII in mediating membrane fusion and exocytosis.

### The linkage between Aging and Lysosomes

**Figure 3.**
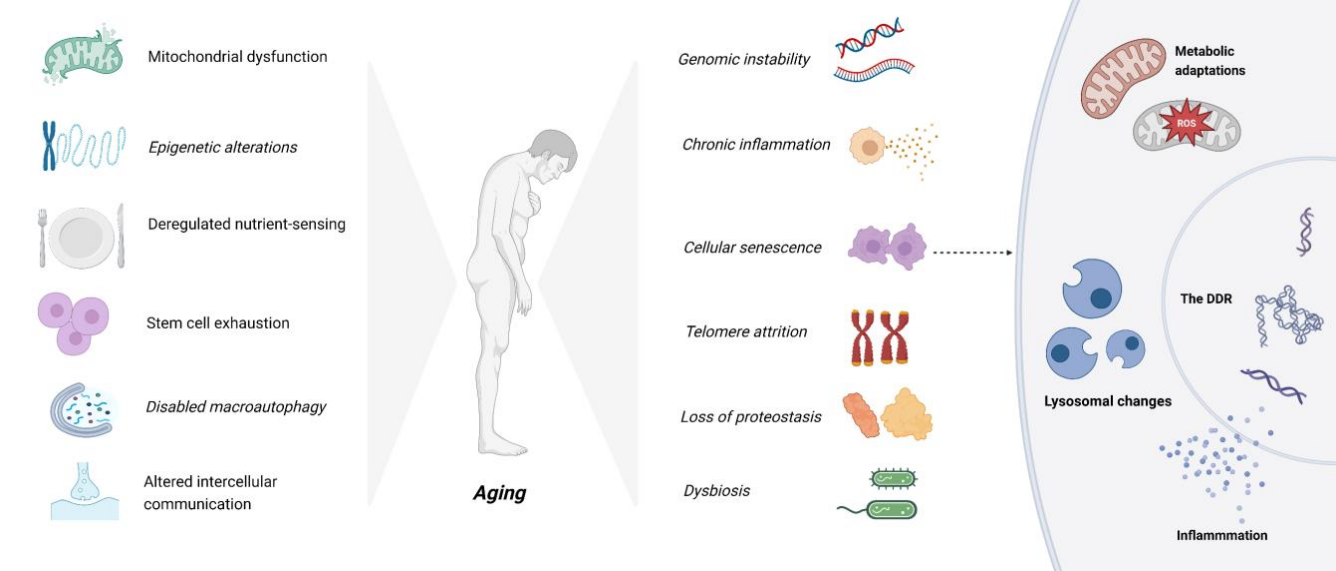
**The hallmarks of aging**. The picture compiles the 12 hallmarks of aging: genomic instability, telomere attrition, epigenetic alterations, loss of proteostasis, disabled macroautophagy, deregulated nutrient-sensing, mitochondrial dysfunction, cellular senescence, stem cell exhaustion, altered intercellular communication, chronic inflammation, and dysbiosis. We also show the major hallmarks of senescence. Created with BioRender.com.

**Figure 4.**
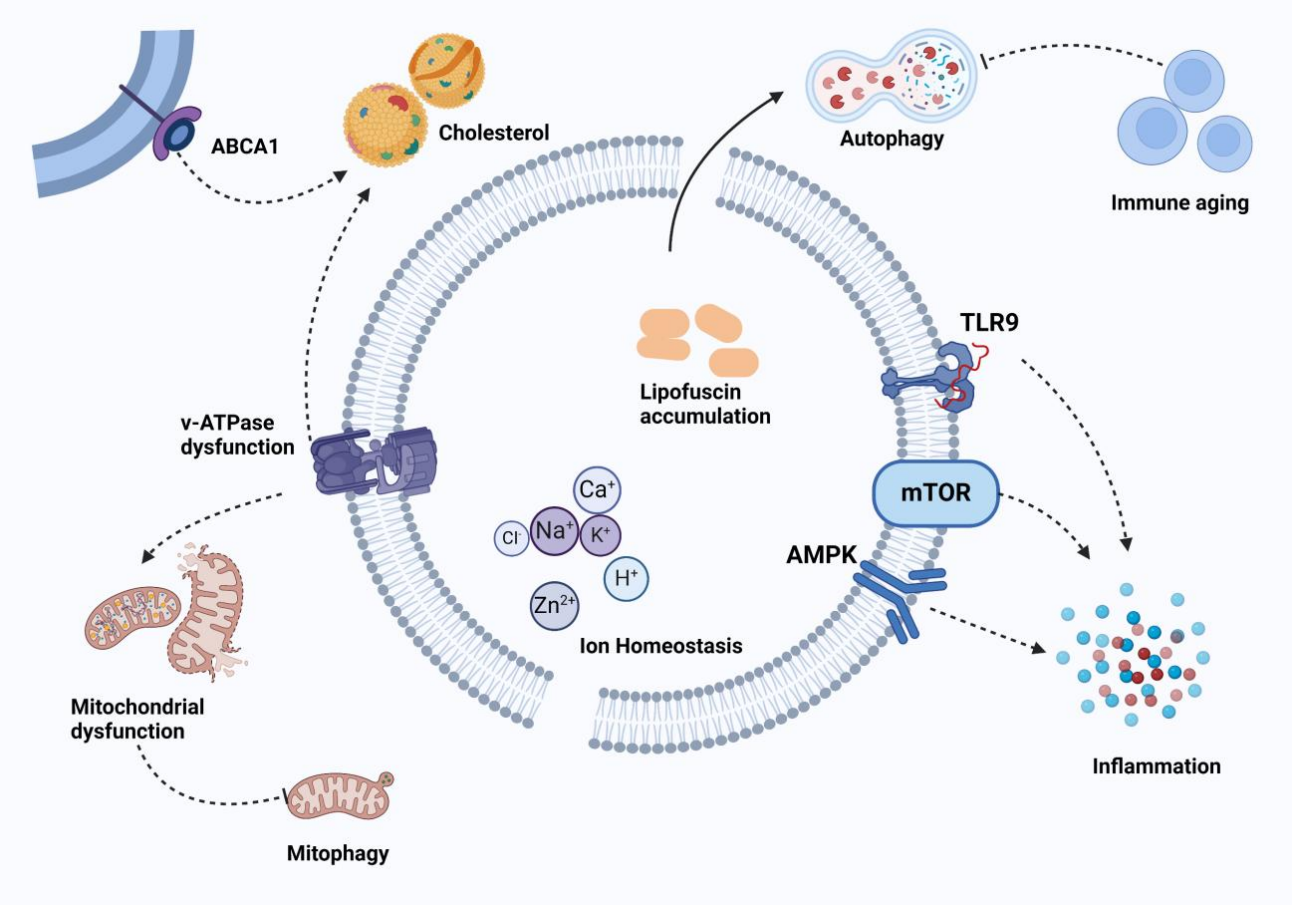
**The linkage between aging and lysosomes**. Lysosomes and ageing are correlated and interact with each other, which includes mitochondrial dysfunction, immune aging, and chronic inflammation. Created with BioRender.com.

Recent studies propose twelve hallmarks of aging [154]. They are shown in Figure 3. The expanding body of knowledge on lysosomal function has led to a more comprehensive understanding of the pathogenic mechanisms underlying lysosomal defects and their association with disease. Specifically, it has been postulated that age-related decline in lysosomal function may account for the increased prevalence of these diseases among elderly individuals [155]. The dysfunction of lysosomes can induce significant cellular alterations, including accumulation of damaged macromolecules and organelles, as well as enhanced secretion from lysosomes [8, 156-158]. The linkage between aging and lysosomes is shown in Figure 4.

### SASP and Inflammation

Senescent cells affect their surroundings by secreting immune-modulatory molecules such as matrix metalloproteinases, growth factors, chemokines, and cytokines. This is known as the senescence-associated secretory phenotype (SASP), which varies depending on cell type and senescence triggers [159]. Senescent cells exhibit lysosomal expansion and dysfunction.

Both mitochondria and lysosomes are associated with SASP production. The depletion of mitochondrial DNA appears to induce a specific SASP pattern known as MiDAS, which lacks the IL-1 inflammatory pathway [160]. In contrast, lysosome disruption appears to activate IL-1, thereby enhancing the functionality of the NLRP3 inflammasome [161]. The upregulation of the lysosomal processing and adaptation system in senescence involves the augmentation of lysosomal exocytosis, which partially contributes to the secretion of proteins associated with the SASP. Proteins secreted by the SASP comprise a wide range of cytokines that collectively play a role in the development of chronic inflammation and tissue damage in different age-related diseases [162]. NLRP3 activation is associated with lysosomal destabilization and the subsequent release of cathepsin B. Additionally, perturbations in cellular integrity can trigger the NLRP3 inflammasome. This process further plays a crucial role in processing pro-IL-1β [161, 163], thereby establishing a connection between inflammation and the compromised integrity of lysosomes. Several authors have shown that treating senescent cells with lysosomal function inhibitors like leupeptin can induce inflammation.

Lysosomes play a key role in controlling the chronic inflammation associated with aging through the modulation of multiple pathways. For example, autophagy influences the immunometabolism of inflammatory immune cells and modulates their differentiation by regulating AMPK and mTOR [164]. Additionally, a decrease in lysosomal function and mitophagy contributes to chronic inflammation and muscle cell atrophy potentially by upregulating Toll-like receptor 9 (TLR9)-mediated inflammation [165]. Senescent CD4^+^ T cells exhibit diminished autophagic activity and reduced expression of LAMP- 2A [166]. Consequently, the diminished autophagic activity in these senescent T cells contributes to chronic inflammation and exacerbates the aging process, ultimately resulting in telomere shortening in T cells [167]. Although the specific contribution of LQC to age-related dysbiosis has yet to be determined, researchers have discovered that autophagy plays an important role in maintaining intestinal balance, regulating gut ecology, supporting immune responses in the intestines, and providing defence against microbial challenges [168].

### Mitochondrial dysfunction

Lysosomes and mitochondria exhibit strong interdependence [169]. The interference of lysosomal function with mitochondrial function during aging through several mechanisms has been confirmed [1]. Mitochondrial damage leads to a decrease in membrane potential, causing inefficient respiratory chain function and ROS production. ROS contributes to the oxidation of proteins and lipids, thereby worsening mitochondrial dysfunction. The elimination of impaired mitochondria is facilitated by macroautophagy, which serves as the primary mechanism for the removal of organelles. Consequently, lysosomes play a direct role in maintaining mitochondrial quality control mechanisms [1]. Mitophagy is the primary link between cellular structures. The association between proper V-ATPase activity and lumen acidification has been linked to deceleration in the aging process observed in yeast [170]. The presence of numerous mutations in the subunits of V-ATPase has also been linked to various neurodegenerative conditions, while animal models harbouring these mutations display problems related to lysosomal acidification along with an expedited aging process [171]. The presence of mitochondrial dysfunctions in cells lacking V-ATPase subunits underscores the significant interplay between lysosomes and mitochondria [172, 173]. Researchers have further suggested that age-related decreases in lysosomal function may be strongly correlated with a decline in mitophagy and the resulting accumulation of dysfunctional mitochondria. This progression ultimately results in the failure of cellular functions [174, 175].

In yeast, aging is associated with the improper retention of neutral amino acids within vacuoles [170]. Furthermore, it has been proposed that the disturbance of amino acid distribution within the lysosome-like vacuole during yeast aging, specifically the hindered storage of cysteine in the vacuole, triggers an increase in cytoplasmic levels of cysteine. The presence of cytoplasmic cysteine restricts iron availability and leads to a decline in mitochondrial respiration function, whereas reducing cysteine levels restores mitochondrial function [176].

### Cholesterol metabolism

Cholesterol is an important component of cell membranes, governing many biophysical and functional properties such as membrane fluidity and the formation of specialized membrane microdomains that serve as signaling centres [177, 178]. Cholesterol further plays a critical role as an essential nutritional component for the mTORC1 pathway and serves as a central controller of cell growth and anabolism. This is achieved through the involvement of lysosomal transmembrane proteins SLC38A9 and LYCHO [35, 36]. As such, it is crucial to implement intricate regulations on cholesterol metabolism in order to prevent the development of various age-related ailments in humans, such as osteoarthritis (OA), Alzheimer's disease, cancer, osteoporosis, and cardiovascular diseases [15, 177, 179-184]. This serves as a cellular foundation for the manifestation of the aging-promoting effects of accumulated cholesterol through the metabolic control of inflammation associated with senescence. The transportation of cholesterol from cells to the lysosome is essential for these actions, emphasizing the crucial role of the lysosome as a signaling hub that governs the cellular direction in response to stress [25, 31]. Modulation of senescence-associated inflammation by cholesterol, with a focus on lysosomes, is a crucial factor in the development of OA, a degenerative joint disease associated with aging [185, 186].

Earlier research indicates that multiple stimuli can accelerate cellular cholesterol metabolism, subsequently triggering senescence initiation [187]. A hallmark of senescent cells is the upregulation of ABCA1, a cholesterol transporter that relocalizes to lysosomes. In this compartment, it acquires a dual function by facilitating cholesterol import [187]. Rewiring this trafficking pathway towards the lysosome aligns with the metabolic requirements for cholesterol, facilitating the maintenance of the mTORC1-SASP axis during senescence. This mechanism is important in osteoarthritis progression. Sustained mTORC1 activation is linked to cancer [115, 188], while the modulation of cholesterol distribution within lysosomes controlled by ABCA1 could potentially serve as a similar mechanism in tumourigenesis [187].

### Immune Senescence

The buildup of cellular components in senescent cells leads to proteostasis dysregulation, a key feature of immune senescence [189]. The efficient functioning of healthy cells relies on advanced mechanisms to remove protein aggregates, impaired organelles, and intracellular pathogens. They employ the autophagy pathway for lysosomal degradation to cope with metabolic and genomic stress, and to rejuvenate during periods of differentiation [116, 190, 191]. Reduced autophagy has been observed in different mammalian tissues with age, including the human brain, murine T cells, and macrophages [192-194]. Aging affects multiple signaling pathways involved in autophagy and the lysosomal compartment, which collectively contribute to impaired autophagy induction and degradation [111]. Indeed, recent evidence has indicated that T cells play an active role in inflammaging [195, 196]. In particular, T cells experiencing mitochondrial dysfunction caused by a lack of mitochondrial TFAM exhibit compromised lysosomal activity, which hastens cellular senescence and inflammaging [196, 197].

Lysosomes, once regarded only as sites for the digestion of endocytosed macromolecules, are now acknowledged as dynamic organelles capable of fusing multiple targets. These entities have thus been recognized as pivotal platforms for nutrient perception and the dissemination of bioenergetic information to the nucleus, thereby assuming critical regulatory roles in determining T cells fate decisions and viability [127]. Additionally, lysosomes are recognized as responsible for secretion, facilitating rapid communication and nutrient transfer between immune and adjacent cells. Lysosomal dysfunction plays a crucial role in facilitating AMPK-mTORC1 communication. Autophagy and lysosomal dysfunction can eventually influence mitochondrial well-being, the efficiency of metabolic adjustments, and the ability of T cells to transform into durable memory T cells, which provide protection against illnesses. Conversely, when these processes go awry, T cells may misdifferentiate into short-lived pro-inflammatory effector T cells, which play a significant role in autoimmune disorders [127].

### Loss of Proteostasis

The collapse of proteostasis, marked by the presence of misfolded, misplaced, or clumped proteins, is a primary characteristic of both aging and disease. However, the loss of proteostasis with age has been observed in various tissues. Further, the accumulation of protein aggregates is firmly associated with neurodegenerative conditions, including amyotrophic lateral sclerosis, Alzheimer's disease, Huntington's disease, and Parkinson's disease [2, 198]. Damaged or misfolded proteins undergo a process called protein quality control that ensures their degradation. The disruption of proteostasis plays a major role in the aging process and the diseases associated with aging. The maintenance of proteostasis relies heavily on the functions of the ubiquitin-proteasome and autophagy-lysosome pathway [199].

Many researchers have demonstrated the importance of lysosomes in the maintaince of cellular proteostasis. Among the different chaperone reactions involved in handling unfolded proteins and preventing proteotoxicity, the heat shock response (HSF) is particularly significant. Additionally, researchers have demonstrated that the induction of HSF-1 in nematodes extends their lifespan, operating via a pathway dependent on lysosomes [200]. Cellular proteostasis is maintained through secretory autophagy, which involves the release of ATG-dependent EVs and particles when conventional autophagic degradation is compromised [201].

### Telomerase activity

In humans, telomere length serves as a predictive indicator of aging, disease, and early mortality. A reduction in telomerase activity linked to the passage of time results in telomere shortening, compromising chromosomal stability. Previous studies have established a direct association between telomerase activity and autophagy [202]. The mTORC1 inhibitor, Rapamycin, partially restores the autophagic response induced by ischemia/reperfusion. This study highlights the link between telomerase and mTORC1 in autophagy activation. Additionally, irisin was found to boost autophagy in aged liver cells by increasing telomerase activity during hepatic ischemia/reperfusion [203]. In contrast, the suppression of hhTERT activity results in an increase in ROS generation and interference with the BECN1/beclin-1 interactions, ultimately compromising autophagy [204]. In addition, hTERT plays an important role in initiating mitophagy after lysosomal damage by regulating the cleavage and subcellular localization of PINK. By inhibiting PINK1 cleavage through the suppression of mitochondrial processing of peptidase β, hTERT enhances the accumulation of PINK1 on the mitochondrial membrane, thereby impeding its transport and elimination of PINK1 in the cytosol [205, 206]. HK2 may serve as a molecular bridge connecting telomerase and autophagy [207]. Telomerase stimulation of HK2 suppresses mTOR activity and triggers autophagy activation. TERC, the RNA component of telomerase, interacts with the 5′-TTGGG-3′ sequence in the HK2 promoter region. These findings suggest the telomerase-HK2-mTOR-autophagy axis is crucial for telomerase-induced autophagy.

### Intercellular communication

Secreted factors such as signaling molecules and extracellular vesicles facilitate long-range intercellular communication in mammals. EVs, including exosomes, microvesicles, and apoptotic bodies, transport biomolecules (lipids, proteins, and nucleic acids), and induce phenotypic alterations in recipient cells. EVs play a role in various physiological processes within the human body. These include the modulation of immune responses, development of neurodegenerative diseases, facilitation of viral spread, and initiation and spread of tumors [208-213]. The involvement of lysosomes in the biogenesis and release of exosomes, which are small lipid-encapsulated particles with diameters ranging from 30 nm to 150 nm, has been established [214].

Emerging findings have indicated that the release of EVs often acts as an alternative mechanism for elimination, circumventing lysosomes [215]. The aging process in long-lived cells, such as cardiac myocytes, leads to an increased demand for lysosomes to remove damaged proteins and organelles. Numerous studies have documented diverse modifications in lysosomes associated with aging, including changes in their size and quantity, as well as compromised activity of lysosomal hydrolases [216]. Previous research has further established an increase in the release of EVs containing mitochondria occurring as a result of compromised internal degradation pathways caused by aging or genetic mutations [217]. The inhibition of lysosomal activity using various alkaline agents has been demonstrated to enhance the secretion of EVs. Treatment with Bafilomycin A increases α-synuclein levels in EVs. This suggests lysosomal inhibition affects α-synuclein release via exosomes [218]. Mutations in tau inhibit lysosomes, leading to increased expression of proteins related to EV secretion [219, 220]. Similarly, concentrations of α-synuclein associated with EVs were shown to be elevated due to mutations in VPS4, a protein essential for the maturation of multivesicular bodies (MVBs) and their fusion with lysosomes [215, 221].

Another example of the enhanced release of EVs due to impaired lysosomal function can be observed in studies conducted on frontotemporal dementia with parkinsonism associated with FTDP-17, which is attributed to the presence of the Tau N279K mutation. It has been shown that flotillin-1 levels are elevated in PPND/FTDP-17 patients with the N279K Tau mutation. NSCs from these patients exhibit impaired endocytic transport, including endosome accumulation, reduced lysosomes, and increased EV markers like flotillin-1 [215, 220].

### The Roles of Lysosomes in Age-Related diseases

**Figure 5.**
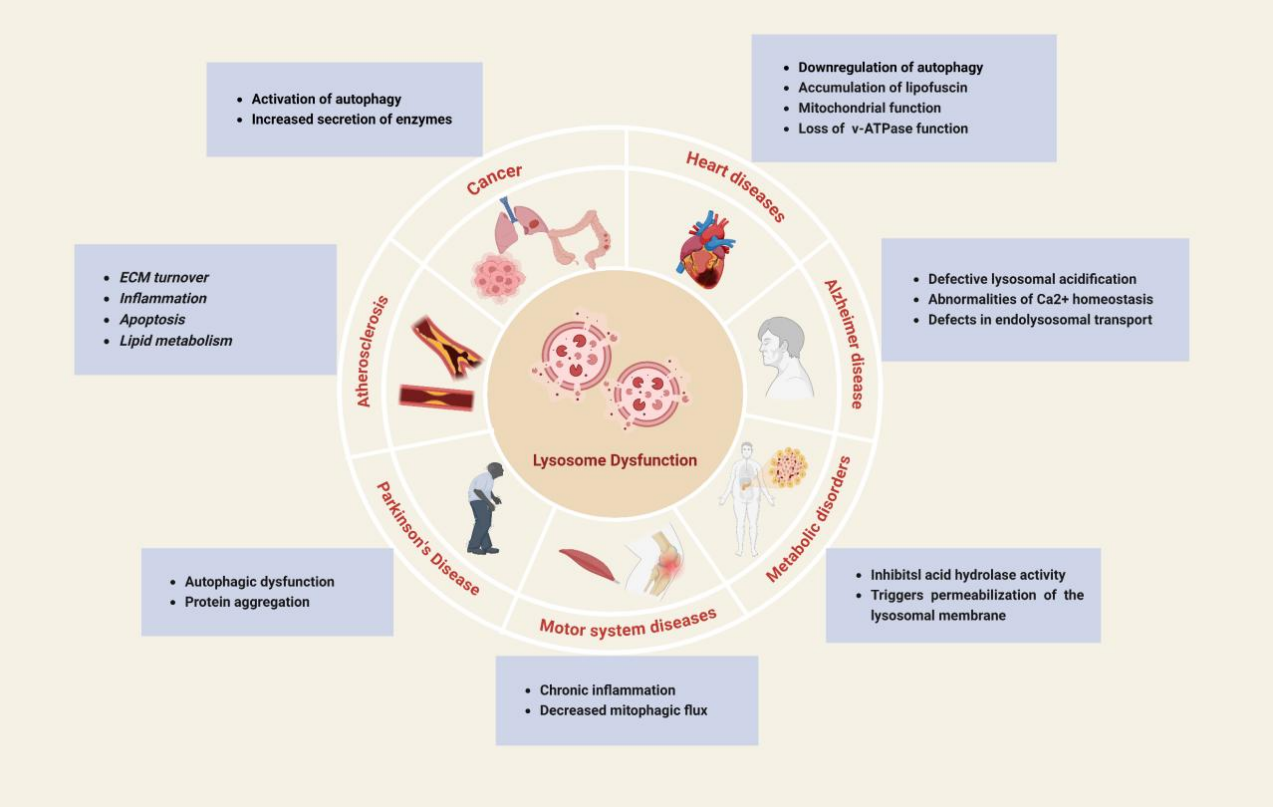
**The correlation between age-related diseases and lysosomes**. Lysosomal dysfunction plays an important role in the development of age-related diseases including cardiovascular diseases, cancer, and neurodegenerative diseases. Created with BioRender.com.

Aging is a natural phenomenon characterized by the gradual decline of cells and bodily functions over time, resulting in a diminished quality of life. Accordingly, aging serves as the main risk factor contributing to the numerous disorders, including cardiovascular diseases, cancer, and neurodegenerative diseases. Age-related illnesses collectively impose a considerable socioeconomic burden on a global scale, posing a significant healthcare obstacle [222, 223]. Hence, it is crucial to identify therapeutic approaches that support “healthy aging”, while effectively preventing the advancement of various age-related diseases and disorders [222]. Figure 5 compiles the correlation between age-related diseases and lysosomes. Emerging data shows lysosomal malfunction affects common conditions like neurodegenerative, metabolic disorders, and cancer, beyond rare hereditary diseases. The lysosome is key for cellular and organismal homeostasis, making it a promising therapeutic target [25].

### Atherosclerosis

The progression of atherosclerosis can trigger the development of coronary syndromes, ischaemic stroke, intermittent claudication, and aneurysms due to plaque growth and thrombus [224-227]. The accumulation of LDL-C and oxLDL within macrophage-derived foam cells in the arterial intima further plays a pivotal role in atherosclerosis, leading to chronic inflammation [228, 229]. Subsequently, these cells undergo programmed cell death, leading to the formation of a necrotic core enveloped by a fibrous cap. Disruption of this cap exposes tissue factors, initiating thrombosis [230]. The development of premature atherosclerosis in humans has been attributed to a significant decrease in the lysosomal acid lipase activity [231]. The process of lipid processing may encounter difficulties when macrophages are exposed to oxLDL [232]. The administration of oxLDL or cholesterol crystals induces lysosomal dysfunction characterized by an elevation in the pH of lysosomes, enhanced permeability of the lysosomal membrane, reduced capacity for degradation, and morphological changes [233]. The role of macrophages as the primary source of foam cells has also been extensively investigated, revealing the crucial involvement of lysosomes throughout the entire process of atherosclerosis [234].

Autophagy is linked to vascular function. Endothelial autophagy dysfunction impairs cell orientation, promotes inflammation, apoptosis, and senescence [235]. Autophagy regulation affects endothelial cell angiogenesis. Silencing Atg5 inhibits tube formation and migration, while upregulating Atg5 promotes autophagy and angiogenesis. Autophagy is also crucial for VSMC plasticity under stress. Regressive changes in ECs or VSMCs lead to fibrous cap attenuation, increasing plaque vulnerability and rupture risk. Autophagy plays a significant role in atherosclerosis progression, as shown by studies on ApoE-null mice with Atg7 deletion in VSMCs. These mice displayed enhanced atherosclerotic plaques formation after 10 weeks on a high-fat diet, as evidenced by the observed increase in cellular apoptosis within the plaque, heightened inflammatory response, and attenuation of the fibrous cap [236]. The lack of autophagy in ApoE-null mice, in which Atg5 is selectively deleted in macrophages, promotes the development of plaques and triggers excessive activation of the macrophage inflammasome, leading to elevated production of interleukin-1β [237]. Collective evidence strongly supports the notion that autophagy dysregulation may play a role in the development of atherosclerosis.

The involvement of cathepsins in cardiovascular diseases, such as atherosclerosis, is attributed to their influence on extracellular matrix turnover, inflammation, and apoptosis [238]. Vascular wall integrity depends critically on extracellular matrix proteins, particularly elastin and collagens. Remodeling the extracellular matrix is considered a fundamental mechanism involved in cardiovascular diseases, given its substantial impact on cellular migration and proliferation within arterial plaques. In contrast to normal arteries, in which cathepsins are minimally or not expressed, these proteins are abundantly secreted and expressed in atherosclerotic vessels [239-242]. The influence of cathepsins on atherosclerosis has further been suggested to be mediated by their effect on lipid metabolism, such as lipoprotein degradation [238].

### Heart diseases

Cardiac myocytes, along with other long-lived postmitotic cells, exhibit significant age-related changes that primarily impact the mitochondrial and lysosomal compartments. Growing evidence has suggested that the age-related buildup of damaged mitochondria may be influenced by various mechanisms, which could involve a reduced likelihood of altered mitochondria being engulfed by autophagy, as well as hindered autophagy due to the excessive accumulation of lipofuscin in lysosomes [243]. With the advent of advanced age, both the composition and structure of the heart and vasculature undergo substantial alterations, resulting in a decline in cardiovascular function [244]. Autophagy mitigates the advancement of contractile dysfunction and remodeling caused by haemodynamic overload by hindering the build-up of misfolded proteins, restoring the proper functioning of mitochondria, and decreasing oxidative stress [245]. The level of cardiac autophagy is diminished below physiological thresholds, which consequently contributes to the advancement of heart failure and occurs during aging [246].

Autophagy serves as a pivotal regulator of aging and lifespan in various organisms, exhibiting a gradual decline in cardiac tissue throughout the aging process. This observation indicates that the downregulation of autophagy is responsible for the advancement of cardiac aging [246]. Essential regulators of autophagy exhibit downregulation in the cardiac aging process. The depletion of NAD+ caused by ROS-induced DNA damage further hampers the Sirt1/FoxO pathway, leading to the downregulation of Atg genes [247]. A decrease in LAMP2 expression, which may indicate a disruption in autophagic flow, also appears to develop during aging [121]. Additionally, the accumulation of lipofuscin in lysosomes is observed during cardiac aging, thereby exerting a suppressive effect on lysosomal activity [246]. The implementation of various interventions aimed at stimulating autophagy not only leads to an extension in life span but also effectively mitigates cardiac aging and enhances stress resistance.

Autophagy is suppressed when there is an accumulation of damaged proteins, as seen in conditions such as DOX-induced cardiomyopathy [248]. In contrast, autophagy exhibits heightened activation under specific circumstances, such as ischemia/reperfusion and the initial stage of pressure overload, potentially contributing to myocardial damage [249]. The maintenance of cardiac function in its initial state depends largely on the vital function of mitophagy, as mitochondria are essential organelles responsible for generating cellular energy and thus play a central role in this process. This is particularly critical for the heart [250]. A decline in mitochondrial function may contribute significantly to age-related alterations in the cardiovascular system [250]. However, during the aging process, baseline cardiac mitophagy is reduced, which is associated with a decrease in mitochondrial function [251]. Mice with parkin specifically deleted cardiac cells rapidly develop fatal heart muscle disease because of the hindered removal of foetal mitochondria through mitophagy. This requires the immediate replacement of adult mitochondria shortly after birth [252]. Similarly, lethal cardiomyopathy caused by the accumulation of foetal mitochondria occurs as a result of the disruption of Mfn2-mediated recruitment of Parkin during weaning [252]. Young mice lacking parkin exhibit a normal cardiac phenotype despite the accumulation of abnormal mitochondria in cardiomy-ocytes with age. Insufficient Drp1 expression inhibits the PINK1/Parkin-mediated mitophagy pathway, resulting in increased myocardial apoptosis in aging hearts. These findings indicate that the PINK1/Parkin signaling pathway likely plays a key role in promoting mitophagy during cardiac aging [250, 253, 254].

Genetic abnormalities of autophagy can lead to cardiomyopathy, while deletion of lamp2 on the X chromosome causes Danon's disease, a condition characterized by hypertrophic cardiomyopathy, skeletal muscle irregularities, and neurological complications. The merging process of autophagosomes and lysosomes, which is crucial for the degradation of autophagic cargo, is impeded by the absence of lamp2b isoform [255]. Mice lacking ragA/B, crucial elements of the lysosomal Ragulator-Rag complex, exhibit compromised lysosomal function because of the absence of v-ATPase activity within lysosomes and decreased autophagic flow, ultimately leading to progressive hypertrophic cardiomyopathy (HCM) [256]. HCM, the primary cardiac manifestation of FD, is a lysosomal storage disorder with an X-linked inheritance pattern resulting from mutations in GLA [257].

### Cancer

The inherent capacity of lysosomes to efficiently eliminate damaged organelles and reuse cellular constituents to sustain the overall health of normal cells also provides benefits to cancer cells. This finding is primarily associated with the observation that triggering lysosome-mediated cell death [81, 84] has proven to be a successful therapeutic strategy for various forms of cancer [258]. In fact, an increase in nutrient-acquiring processes such as autophagy, macropinocytosis, and lysosomal activity has been recognized as a significant factor in the advancement and spread of cancer [259-261]. The growth of various cancers has further been shown to rely on the constitutive activation of autophagy in various in vitro and in vivo model systems [262]. MiT/TFE transcription factors exhibit a unique ability to coordinate catabolic pathways, which are critical for stress response and nutrient recycling, alongside anabolic processes in multiple cancer types such as lung carcinoma, renal cell carcinoma, and melanoma [263]. Autophagy captures and targets large molecules for degradation. It has dual roles in cancer, promoting or suppressing tumors depending on the context [259, 264].

The involvement of lysosomes extends beyond their role in cancer cell proliferation and involves the processes that facilitate invasion and metastasis. In the presence of an acidic tumor microenvironment, lysosomes tend to relocate to the outer region of cells [148]; this relocation process may potentially enhance cell proliferation by enhancing mTORC1 and mTORC2 signaling [265, 266]. Additionally, it could facilitate invasion and metastasis via the excretion of lysosomal hydrolases [267], matrix metalloproteinase, and integrins [268, 269]. The potential for the induction of angiogenesis, promotion of tumor growth, and facilitation of invasion may further be attributed to the release of cathepsins with proteolytic activity into the extracellular environment [270]. Cathepsin D facilitates the extracellular functions of cathepsins by degrading cystatin C, which is a natural inhibitor of cysteine cathepsins [271]. Additionally, M6PR specifically targets secreted lysosomal enzymes, and is frequently subject to mutations or downregulation in a diverse array of malignant tumors, leading to augmented secretion of lysosomal enzymes. The forced presence of M6PR can further reduce the ability of tumor cells to form tumors and invade by restoring the movement of cathepsins within cells towards lysosomes [272]. Targeting lysosomes could also potentially serve as a viable therapeutic strategy to prevent or delay the development of therapy resistance, as well as to enhance the efficacy of anticancer drugs across various tumor contexts [264].

### Neurodegenerative diseases

Lysosomal dysfunction is associated with several prevalent neurodegenerative disorders [273], encompassing Parkinson's disease, Alzheimer's disease, Huntington's disease, and the most prevalent neuromuscular disease known as Charcot-Marie-Tooth disease [274-278].

### Alzheimer disease

The characteristic features of AD include the presence of Aβ peptides in amyloid plaques outside cells, concurrent with the build-up of neurofibrillary tangles inside cells [279]. It has been suggested that the malfunction of the autophagy-lysosome pathway may play a significant role in AD [280]. Indeed, risk alleles associated with the onset of Alzheimer's disease at a later stage, such as TREM2, APOE, PLD3, BIN1, CD2AP, PICALM, and SORL1, have all been associated with pathways related to lysosomal autophagy. Additionally, these genes are involved in APP processing, a pathway that is both genetically and pathologically relevant in Alzheimer's disease [281-284]. Lysosomal dysfunction in glial and neuronal cells promotes the spread of pathological markers in AD and PD [282, 285, 286]. Collectively, these results highlight the widespread impact of lysosomal dysfunction on diverse cell types within the nervous system.

Patients diagnosed with AD and possessing mutations in PSEN1 exhibit impaired lysosomal acidification and disrupted Ca^2+^ homeostasis, resulting in compromised autophagy and lysosomal function [287-289]. Additionally, research has shown that impairments in endolysosomal transport led to modifications in the production of amyloid-β peptides in AD [25, 290]. In AD, amyloid plaques exhibit a significant accumulation of lysosome-like organelles. These structures display diminished protease levels, indicating defective maturation. Notably, they localize preferentially at sites where swollen axons interact with amyloid deposits [291].

### Parkinson disease

A considerable proportion of individuals diagnosed with PD harbour mutations in lysosomal-autophagic genes [292, 293]. Lysosomal autophagic dysfunction is linked to protein aggregation in neurodegenerative disorders. In Parkinson's disease, this dysfunction leads to α-synuclein accumulation in Lewy bodies [25, 294, 295]. Lysosomes possess the capacity to facilitate the bidirectional transport of ions across their membranes, thereby ensuring internal homeostasis, and initiating downstream signaling cascades. Lysosomes are further equipped with a diverse array of ion channels that have direct implications in human diseases. In individuals with PD, as well as in the associated disorders DLB and RBD, TMEM175 has been found to harbour genetic variations that confer both susceptibility and protection [296-298]. The lysosomal patch-clamp technique revealed that TMEM175 encodes a potassium channel localized within the lysosome. Furthermore, protective and risk variants associated with Parkinson's disease can either activate or inactivate this channel [73, 299]. Notably, separate research revealed that this protective genetic variant did not merely enhance channel activity but also strengthened neuronal resistance to injury [299]. Based on these results, the activation of TMEM175 could serve as a potential promising therapeutic approach for PD [282].

### Metabolic disorders

Metabolic disorders like T2DM, insulin resistance, and sarcopenic obesity are rising in elderly populations, causing global concern [300]. The process of aging induces various physiological alterations in body composition, leading to an augmentation in adipose tissue mass, particularly visceral fat accumulation, as well as a decline in skeletal muscle mass and strength; these modifications are closely associated with metabolic disorders [301, 302]. Lysosomes have further been involved in the progression of several metabolic conditions, particularly obesity and diabetes [303-305]. Obesity-associated lipid overload thus hampers lysosomal function via multiple mechanisms [303]. For example, the consumption of a diet rich in fats by mice hinders the acidification process and the activity of acid hydrolases within lysosomes, leading to the disruption of the lysosomal membrane, resulting in impaired autophagy and lysosomal function across various tissues [306]. Insulin resistance and β-cell dysfunction are the primary factors leading to TD2, while dysregulated autophagy has been identified as a significant contributing factor [307]. Studies have detected increased autophagosome accumulation in both rodent β-cells and diabetic animal models. Similarly, pancreatic β-cells isolated from type 2 diabetes patients exhibited numerous autophagic vacuoles [308]. A potential causal mechanism has been suggested, in which chronic glucolipotoxicity leads to lysosomal defects, resulting in the obstruction of β-cell autophagic flux [309].

Obesity and T2D are commonly linked with NAFLD, which is among the most prevalent chronic liver disorders in Western countries. Numerous genetic interventions have provided compelling evidence to support the crucial role of autophagy in the liver and metabolic disorders. Mice with targeted removal of Atg7 from their hepatocytes exhibited liver enlargement and significantly elevated levels of triglycerides and cholesterol, resembling the characteristics of NAFLD [310]. NAFLD ranges from the presence of fat in the liver (non-alcoholic steatosis, NAS) to inflammation and damage to the liver due to fat accumulation (nonalcoholic steatohepatitis, NASH), which can subsequently progress to fibrosis, cirrhosis, and impaired liver function. Emerging findings from human research have suggested that hepatic autophagy is compromised in individuals with biopsy-confirmed NAS or NASH, as opposed to those with a liver histologically classified as normal. Notably, hepatic mRNA expression of ER stress markers (GRP78 and CHOP) and autophagy-related genes is significantly upregulated in NASH patients relative to NAS controls. This pattern is mirrored at the protein level, with NASH specimens demonstrating substantially increased expression of GRP78, CHOP, and p62/SQSTM1 compared to NAS tissues [311]. The upregulation of Rubicon, a negative regulator involved in the fusion of autophagosomes and lysosomes, has been observed in the livers of mice subjected to HFD, as well as in individuals diagnosed with NAFLD, suggesting compromised autophagy [312].

Several metabolic disorders, including sarcopenic obesity, insulin resistance, and T2DM, are strongly associated with autophagy dysfunction. Hence, the promotion of autophagy could serve as a potential therapeutic approach to improve age-related metabolic disturbances. With aging, there are significant alterations in the quantity, arrangement, cellular makeup, and hormonal communication within the adipose tissue. These alterations have been associated with the development of insulin resistance and metabolic dysfunction [313]. Patients with obesity or T2DM exhibit increased autophagy in both subcutaneous and visceral adipose tissue [314-317]. In addition, the decline in RUBCN expression observed in adipose tissue with advancing age led to an increase in autophagic activity. The adipocyte-specific knockout of Rubcn in mice resulted in reduced fat tissue, impaired glucose tolerance, abnormal lipid levels, and accumulation of fat in the liver. This is caused by a decrease in the ability to store lipids and a decline in the endocrine function of adipokines such as adiponectin. Hence, rubicon plays a crucial role in preserving the functionality of adipocytes, as well as maintaining the overall metabolic equilibrium by restraining excessive autophagy [301].

### Motor system diseases

Age-related phenotypes encompass a spectrum of conditions, including osteopenia (defined as a decrease in bone mass), sarcopenia (a decline in cartilage and muscle mass), frailty, and multimorbidity, which exhibit varying rates of progression among individuals over time. Sarcopenia has further been characterized by a gradual decline in muscle mass accompanied by aging. The onset of sarcopenia is linked to chronic inflammation resulting from impaired lysosomal function and reduced mitophagic flow associated with advancing age [165]. Aging tissues also show a gradual accumulation of lipofuscin, commonly known as "the age pigment," which serves as an indicator of impaired lysosomal clearance [318]. Disturbances in the lysosomes function in aged muscles may have significant implications for autophagy and mitophagy. Evidence has suggested an increase in the levels of FoxO3 and TFEB proteins in aged muscles, which supports the increased expression of genes related to autophagy and the ubiquitin-proteasome system in older muscles [319]. However, whether the phosphorylation and localization of FoxO3 are influenced by age in relation to AKT activity currently remains unclear [320]. Furthermore, mTORC1 activity is sustained in the aged muscle [321].

The severe cardiac and skeletal muscle myopathy observed in Glycogen Storage Disease Type II has been attributed to the progressive accumulation of glycogen caused by inadequate levels of lysosomal acid alpha-glucosidase [322]. However, the correlation between osteoporosis and lysosomal function remains unclear Gasdermin D (GSDMD) facilitated pyroptosis modulaters immunogenic cell death and inflammation. Li et al. discovered that GSDMD plays a crucial role in the regulation of lysosomal maturation and exocytosis in osteoclasts. The deletion of GSDMD results in the development of severe osteoporosis associated with aging and characterized by significant trabecular bone loss [323]. TET2, a protein known as Tet methylcytosine dioxygenase 2 enzyme that removes methyl groups from DNA. Its function involves facilitating osteoclast differentiation by suppressing BCL2 and enhancing autophagy through the upregulation of BECN1-dependent mechanisms [324].

### Emerging therapy strategies targeting lysosomes

The process of aging involves a range of modifications in the functionality and structure of lysosomes. Although certain alterations can be beneficial, they may also accelerate the aging process and contribute to diseases associated with old age [206]. We show potential therapeutics targeting lysosomes for the treatment of age-related diseases in Table 2.

### Regulating autophagy

The autophagic system is essential for degrading cytoplasmic components, from individual proteins to entire organelles [7]. This degradation process requires functional coordination with lysosomes for effective clearance. Notably, autophagy significantly contributes to disease pathogenesis across multiple conditions - including atherosclerosis, neurodegenerative disorders, autoimmune diseases, and cancer-with particular importance in modulating macrophage polarization. As previously stated, autophagy activation by Latrepirdine and CCI-779 has been identified as a potential therapeutic approach for PD [325]. This intricate cellular process offers numerous potential avenues for intervention. Among the proteins involved in the autophagy regulatory pathway, mTOR is one pivotal target. Indeed, numerous clinical trials have investigated the application of mTOR inhibitors, including temsirolimus, sirolimus, and rapamycin, either as standalone treatments or in conjunction with other therapies, for conditions such as atherosclerosis, autoimmune disorders, and malignant tumors. There is a consensus that mTOR inhibition promotes autophagy.

Strategies aimed at enhancing autophagic activity have been suggested as potential strategies for eliminating senescent cells. Specifically, the inhibition of mTORC1 has been demonstrated to prolong lifespan in various animal models, leading to the development of inhibitors, such as rapamycin or torin1, targeting this pathway [326]. Autophagy has further been demonstrated to be enhanced while inflammation is improved by various other molecules, including metformin [327]. The inhibition of mTORC1 hinders geroconversion, which refers to the shift from a dormant state to senescence, highlighting the significance of mTORC1 activity in senescence [328]. The administration of melatonin facilitates fusion between mitophagosomes and lysosomes, thereby augmenting mitophagy, leading to the restoration of mitochondrial functions, a reduction in Aβ pathology, and an improvement in cognitive abilities [329].

Multiple studies have demonstrated that the virus-mediated overexpression of TFEB elicits a remarkable augmentation in lysosomal biogenesis and autophagy. This intricate process culminates in the eradication of substances accumulated within cells, thereby substantially ameliorating the disease phenotype observed in murine models of neurodegenerative disorders [330]. TFEB expression declines with age, while the restoration of TFEB levels can delay senescence in various models [118, 331]. Given the pivotal role of TFEB in regulating lysosomal biogenesis, numerous efforts have been undertaken to discover enhancers that may facilitate lysosomal activity and autophagy, as optimal lysosomal function is crucial to ensure longevity. The activation and upregulation of TFEB by numerous compounds occur downstream of mTORC1 inhibition, and a specific compound, C1, has been identified as a direct activator of TFEB. Consequently, C1 binds TFEB, and facilitates its translocation into the nucleus [332].

Importantly, previous studies have shown that improving mitophagy has significant potential as a therapeutic strategy for mitochondrial muscle disorders. For example, autophagy-related genes such as LC3 and Bnip3 are regulated by FOXO3. As such, targeting FoxO3 and Bnip3 to modulate autophagy has potential therapeutic applications in muscle wasting disorders and various degenerative ailments [333]. Physical activity triggers BCL2-controlled autophagy benefits muscle glucose homeostasis, and protects the human body from diabetes and other metabolic disorders [334]. Moreover, in osteoporosis, the osteogenic differentiation capacity of BMSCs is hindered primarily by the decreased expression of TP53INP2. This downregulation is induced by oxidative stress and mediated by autophagy, leading to the degradation of TP53INP2 [335].

### Targeting ion channels

Intracellular concentration can be manipulated to enhance lysosomal degradation. The optimal pH within the lysosomal compartment is crucial for proper organelle function, as it governs the release of cargo, maturation of hydrolases, degradation processes, autophagy mechanisms, and intracellular transportation [336, 337]. In conditions characterized by abnormal lysosomal pH, the implementation of therapeutic measures to restore proper acidification within these organelles may offer a promising strategy to facilitate the breakdown and removal of accumulated macromolecules [258]. The optimisation of lysosomal acidification can help to maintain the optimal activity of lysosomal hydrolytic enzymes. Elevated expression of V-ATPase subunits can effectively enhance lysosomal acidification, leading to various health benefits, such as improved motor activity in mouse models of PD and extended lifespan in yeasts [338, 339]. In contrast, TFEB overexpression or activation has the potential to increase lysosomal acidification by promoting the transcriptional upregulation of genes associated with lysosomes such as V-ATPase subunits.

TRPML1 not only activates TFEB through Ca^2+^ signaling, but also acts as a transcription target of TFEB [340], thereby establishing a positive feedback loop in this lysosomal stress response. Similarly, TFEB overexpression promotes lysosomal substrate clearance through TRPML1-mediated exocytosis [341]. TRPML1 chemical agonists have been demonstrated to enhance lysosomal exocytosis, offering protection against NPC disease [342]or a-synuclein toxicity [343]. Progression of Parkinson's disease is linked to the activation of senescence-induced lysosomal exocytosis through TNF-α, which facilitates the spread of α-synuclein [344]. This is particularly relevant in synucleinopathies such as PD, where SNARE-dependent lysosomal exocytosis contributes to the release of pathogenic α-synuclein species from neurones. These results suggest that this process may contribute to the propagation of Syn pathology via interneuronal transmission [345]. Lysosomal function relies heavily on the maintenance of an optimal pH level. TMEM175, located on the surface of lysosomes, acts as a channel activated by protons and selectively allows the leakage of H+ ions from lysosomes. In the context of Parkinson's disease, studies have revealed that a deficiency in TMEM175 leads to the excessive acidification of lysosomes consequently impairing protease activity and facilitating the aggregation of α-synuclein [346]. Dysfunctional synaptic vesicle endocytosis, regulated by PD-related genes such as LRRK2, PRKN, and VPS35, contributes to disease pathogenesis [347]. The dynamic establishment membrane contact sites between the mitochondria and lysosomes facilitates reciprocal communication and governs their functions [19]. The dynamic and transient membrane contact sites between mitochondria and lysosomes facilitate the bidirectional communication and regulation of their respective functions. Abnormalities at these contact sites have been observed in various neurodegenerative disorders, including Charcot-Marie-Tooth Disease, Parkinson's disease, and Niemann-Pick disease type C [348]. Ongoing preclinical and clinical studies present compelling data supporting the effectiveness, safety, and favourable tolerability of DNL201, a leucine-rich repeat kinase 2 inhibitor, in restoring lysosomal function in individuals diagnosed with Parkinson's disease, without any unfavourable observations [349].

### Regulating the activity of the cathepsin

The mild inhibitory effects of PADK, specifically targeting cathepsins B and L, results in the augmentation of hydrolase synthesis and maturation at lower concentrations, consequently leading to increased lysosomal clearance capacity [350]. PADK treatment in an AD transgenic mouse model increases cathepsin B levels, both in protein expression and activity. It also clears intracellular amyloid-beta deposits and reduces extracellular plaque formation [351].

**Table 2:** Potential therapeutics targeting lysosomes for the treatment of age-related diseases,

Intervention and therapy	Mechanism	Disease	References
**Metformin**	Activate autophagy ActivateTAK1-IKKα/β and AMPK	AD	[359, 360]
**Memantine**	Activate autophagy mTOR inhibition	AD	[361]
**DNL201**	Inhibition of LRRK2	PD	[349]
**Sarcosine**	Activate autophagy Activate AMPK	PD	[362]
**PLEKHM1 Activation**	Lysosomes trafficking	Osteopetrosis	[363]
**Disaccharide trehalose**	Activate TFEB	AS	[364]
**Infusion of recombinant human LAL**	Decrease of LAL activity	AS	[365]
**Gypenoside XVII (GP-17)**	Activated TFEB Enhanced lysosome biogenesis Activate autophagy	AD	[366]
**Farnesyl transferase inhibitors**	Enhanced lysosome activity	TAU-related neuropathy	[367]
**Imanixil/SAR088**	Enhanced autophagosome- lysosome fusion	Diabetes	[368]
**Latrepirdine**	Activate autophagy	PD; HTT	[325, 369]
**CCI-779**	Activate autophagy	PD; HTT	[325, 369]
**Temsirolimus**	Activate autophagy inhibition of mTOR	AS	[370]
**Rapamycin**	Activate autophagy inhibition of mTOR	AS	[370, 371]
**Sirolimus**	Activate autophagy inhibition of mTOR	AS	[370, 372]
**Trehalose**	Inducing autophagy	AD	[234]
**Hydralazine**	Inducing autophagy	AD	[326]

Abbreviations: AD, Alzheimer's disease; AMPK, AMP-activated protein kinase; mTOR, mammalian target of rapamycin; AS, Atherosclerosis; PD, Parkinson's Disease; HD, Huntington's disease; TAK1, ransforming growth factor beta-activated kinase1; TFEB, transcription factor.

The early occurrence of pancreatic acinar cells that simultaneously contain lysosomes and digestive enzymes is regarded as a phenomenon of their coexistence during the development of acute pancreatitis. Trypsin activation leads to the cytoplasmic release of cathepsin B and subsequent lysosomal leakage, triggering the initiation of cellular apoptosis and necrosis. Ultimately, this process triggers pancreatic injury via activation of cell death pathways. Related studies have indicated that the preservation of lysosomal structure and function, as well as the suppression of cathepsin B activity, could potentially serve as effective therapeutic targets for acute pancreatitis [352]. A number of inhibitors targeting cathepsins are currently under development to treat autoimmune diseases, including systemic lupus erythematosus, rheumatoid arthritis, and Sjögren syndrome. These disorders exhibit elevated levels of cathepsin expression or activity owing to factors such as abnormal pH in the lysosomal lumen. Many ongoing clinical trials are being conducted to assess the efficacy of these inhibitors [353].

The intriguing potential of cathepsins as targets for cancer therapy lies in their remarkable pro-neoplastic characteristics, particularly when released into the extracellular environment [354, 355]. The repression of cathepsins could be advantageous in cancer treatment; however, the simultaneous inhibition of multiple cathepsins is necessary because of their overlapping functionality. Targeting lysosomal exocytosis may be an effective therapeutic strategy. Furthermore, optimal lysosomal function relies heavily on maintaining an appropriate lysosomal pH level. Inhibition of vacuolar ATPases effectively prevents the invasion of human prostate cancer cells [356]. In six distinct cell lines of head and neck squamous cell carcinoma, the response to cisplatin treatment correlated with the pH levels within lysosomes [357]. The response of cells to cisplatin was enhanced through a reduction in lysosomal pH via trichostatin A treatment [358].

## Discussion

As an important organelle in cells, lysosomes are involved in cell signal transduction, metabolism, growth, apoptosis, autophagy, protein processing and maintenance of cell homeostasis. Their dysfunction is associated with a variety of aging-related diseases including lysosomal storage diseases, cancer, cardiovascular diseases, and neurodegenerative diseases. At present, there are many emerging therapies for lysosomal disorders, including, enzyme replacement therapy, gene therapy, transplantation and lysosomal drug targeting, but many of them have not yet been validated [373].

Several clinical studies have demonstrated that mTOR inhibitors, including temsirolimus, sirolimus, and rapamycin, can be employed either as standalone treatments or in conjunction with other therapeutic approaches to address atherosclerosis, autoimmune disorders, and various malignancies. Compounds such as trehalose and hydralazine might offer potential benefits for AD by triggering autophagy (as seen in trials NCT04663854 and NCT04842552). Additionally, compounds such as bafilomycin A1, chloroquine, and 3-methyladenine have been shown to intricately regulate autophagic processes within macrophages. This modulation not only amplifies pro-inflammatory signals but also attenuates the expression of anti-inflammatory markers, thereby orchestrating a delicate balance in immune responses. Such a mechanism may serve as a pivotal foundation for advancing clinical research focused on specific cancer types [374-377].

Moreover, studies have shown that CVDs can be improved by activating TFEB. In ApoE KO mice, trehalose decreased the development of atherosclerosis and enhanced cardiac remodeling following myocardial infarction [378, 379]. Autophagy is induced in the hearts of mice by treatment with 3,4-dimethoxychalcone, leading to improved myocardial infarction outcomes following ischaemic injury [380]. The FDA-approved cyclodextrin derivative, 2-Hydroxypropyl-β-cyclodextrin, currently utilized to enhance the solubility of lipophilic drugs, has been identified as a TFEB activator capable of augmenting the autophagic clearance of intracellular proteolipid aggregates. The activation of TFEB has further been demonstrated to have an advantageous impact on metabolic disorders. Another study has highlighted the inhibitory effects of TFEB activation on metabolic disorders. The compound MSL, also known as 4-sulfonyl-5-methylthio-2-phenyloxazole, was discovered to activate TFEB and effectively ameliorate adipose inflammation, hyperglycaemia, hepatic steatosis, and obesity in diabetic mice with the ob/ob phenotype [381, 382].

Despite the development of numerous cathepsin inhibitors in pre-clinical studies, only a limited number, like Odanacatib, have progressed to clinical trials. However, the efficacy of these inhibitors is not consistently satisfactory. This may result from the disruption of critical physiological functions of cathepsins or due to non-specific inhibitory effects [234, 383]. Notably, Odanacatib, the sole selective inhibitor that reached Phase III clinical trials, was halted because of side effects associated with strokes [384]. Future advancements in the development of cathepsin inhibitors may necessitate the incorporation of targeted delivery mechanisms, localized administration strategies, and integration with complementary therapeutic approaches [234].

Novel treatment modalities such as endosymbiont therapy, artificial lysosome delivery, and lysosomal transplantation offer promising avenues for managing lysosomal storage disorders. Despite the great potential of these treatments, numerous challenges remain in terms of safety and ethical implications [373]. In this review, we elucidate the pathophysiological role of lysosomes in aging-related diseases. Lysosomes maintain human homeostasis by critically regulating immunity, metabolism, proliferation, and apoptosis. Consequently, targeting lysosomes in specific human diseases represents a promising and feasible strategy for exploring potential therapeutic interventions.

## Conclusion

Lysosomal dysfunction is linked to aging and age-related diseases, but the exact mechanisms driving its role in aging, cellular senescence, and disease progression are still unclear. In this review, we comprehensively summarized the physiological functions of lysosomes, elucidated their roles in various age-related diseases, and explored potential therapeutic approaches to target these organelles. Furthermore, we identified several hallmarks of aging closely associated with lysosomal dysfunction. A detailed understanding of the biochemical and molecular intricacies underlying these physiological processes not only provide valuable insights into disease pathogenesis, but also facilitates the development of novel diagnostic tools, and the design of innovative treatment agents. Given their pivotal involvement in numerous cellular processes, lysosomes can be considered promising targets for therapeutic interventions. Manipulating lysosomal function through future strategies holds great promise for treating common diseases.

## Future Perspectives

In this review, we provide an overview of the physiological roles of lysosomes, their involvement in several age-related diseases, and possible therapeutic strategies that target lysosomes. While numerous studies illuminated the roles of lysosomes, there remain several mechanisms that continue to elude our understanding. In summary, growing evidence has underscored the pivotal role of lysosomes as crucial organelles in disease pathogenesis. A more comprehensive comprehension of lysosome-associated mechanisms would greatly promote the advancement of targeted therapies aimed at modulating lysosomal function. A deeper understanding of lysosome processes governing cellular homeostasis, as well as the impact of lysosome biogenesis and turnover on these functions, would greatly facilitate the advancement of treatment approaches targeting lysosome-related diseases.
